# An ensemble of the iCluster method to analyze longitudinal lncRNA expression data for psoriasis patients

**DOI:** 10.1186/s40246-021-00323-6

**Published:** 2021-04-20

**Authors:** Suyan Tian, Chi Wang

**Affiliations:** 1 Division of Clinical Research, The First Hospital of Jilin University, 1 Xinmin Street, Changchun, Jilin, 130021 People’s Republic of China; 2 Department of Internal Medicine, College of Medicine, University of Kentucky, 800 Rose St, Lexington, KY 40536 USA; 3 Markey Cancer Center, University of Kentucky, 800 Rose St, Lexington, KY 40536 USA

**Keywords:** Psoriasis, Long non-coding RNAs (lncRNAs), Longitudinal data, Integrative clustering (iCluster)

## Abstract

**Background:**

Psoriasis is an immune-mediated, inflammatory disorder of the skin with chronic inflammation and hyper-proliferation of the epidermis. Since psoriasis has genetic components and the diseased tissue of psoriasis is very easily accessible, it is natural to use high-throughput technologies to characterize psoriasis and thus seek targeted therapies. Transcriptional profiles change correspondingly after an intervention. Unlike cross-sectional gene expression data, longitudinal gene expression data can capture the dynamic changes and thus facilitate causal inference.

**Methods:**

Using the iCluster method as a building block, an ensemble method was proposed and applied to a longitudinal gene expression dataset for psoriasis, with the objective of identifying key lncRNAs that can discriminate the responders from the non-responders to two immune treatments of psoriasis.

**Results:**

Using support vector machine models, the leave-one-out predictive accuracy of the 20-lncRNA signature identified by this ensemble was estimated as 80%, which outperforms several competing methods. Furthermore, pathway enrichment analysis was performed on the target mRNAs of the identified lncRNAs. Of the enriched GO terms or KEGG pathways, proteasome, and protein deubiquitination is included. The ubiquitination-proteasome system is regarded as a key player in psoriasis, and a proteasome inhibitor to target ubiquitination pathway holds promises for treating psoriasis.

**Conclusions:**

An integrative method such as iCluster for multiple data integration can be adopted directly to analyze longitudinal gene expression data, which offers more promising options for longitudinal big data analysis. A comprehensive evaluation and validation of the resulting 20-lncRNA signature is highly desirable.

**Supplementary Information:**

The online version contains supplementary material available at 10.1186/s40246-021-00323-6.

## Background

Psoriasis is an immune-mediated, inflammatory disorder of the skin with chronic inflammation, and hyper-proliferation of the epidermis [1]. It is well supported that psoriasis has genetic components. Based on this feature and because the diseased tissue is very easily accessible, it is natural to characterize the molecular profiles of psoriasis, and thus to investigate its pathogenesis and to develop its targeted immune therapies with the aid of high-throughput technologies.

Long non-coding RNAs (lncRNAs) are post-transcriptional and epigenetic regulators that have lower expression levels and are more tissue-specific compared with protein-coding genes [2]. Once regarded as evolutionary junk, lncRNAs are now known to play essential roles in many complex diseases, especially in cancers [2]. However, their implication in psoriasis has been rarely investigated and remains poorly understood. Among the limited amount of research carried out to explore the roles that lncRNAs may play in psoriasis, some encouraging results show that lncRNAs are of essential importance in this disease. For example, a very recent study [3] genotyped single nucleotide polymorphisms (SNPs) of antisense non-coding RNA in the INK4 locus (*ANRIL*) in 286 patients with psoriasis and 300 controls, and demonstrated that this lncRNA can be regarded as a risk locus of psoriasis. Another study [4] showed maternally expressed gene3 (*MEG3*), a competing endogenous RNAs (ceRNA) of miR-21, was significantly downregulated in lesional skin of psoriasis. Furthermore, by carrying out the weighted gene correlation network analysis (WGCNA) [5], a study [6] suggested that in psoriasis, instead of acting alone many lncRNAs functioned coordinately to impact its onset, progression, and treatment.

Transcriptional profiles not only vary under different conditions or in different tissues but also change correspondingly as a disease initializes and advances, or after an intervention or a stimulus. Unlike cross-sectional gene expression data (expression levels measured at a single time point for each individual), longitudinal gene expression data can capture such dynamic changes and infer the causality relationship between these temporal changes and the phenotype of interest. Consequently, the amount of such data has increased dramatically. For psoriasis alone, several longitudinal gene expression data [7–10] have been stored in the GEO database, which provides researchers a unique opportunity to explore psoriasis deeply from different points of view. In [8], for example, longitudinal gene expression profiles obtained pre-treatment and at intermediate time-points were used to predict the response of individual patients with psoriasis to immune treatments. After evaluating the predictive accuracy of response status using single time point and longitudinal data, it is concluded that the gain in predictive accuracy resulting from including additional time points is substantial.

In this study, a medium-sized longitudinal dataset [8] was reanalyzed using the iCluster method [11], an integrative clustering method that combines multiple omics data for better characterization and segmentation of a specific disease. The objective of this study is to identify crucial lncRNAs which can explain the dynamic differences in between the responders and the non-responders to a specific treatment, judged by the PASI75 index, an indicator of whether at least 75% reduction of the Psoriasis Area & Severity Index (PASI) has been achieved for 12 weeks or longer after being treated.

The iCluster method [11] was proposed by Shen et al. to integrate multiple big genomics data together and thus cluster the samples by using a joint latent variable model. Subsequently, in order to eliminate or alleviate drawbacks of the iCluster method (for example, the original version can only model continuous genomic data), the method itself has been updated or extended to several versions since its initiation, e.g., iClusterPlus [12], iClusterBayes [13], an iCluster extension that explicitly includes an extra penalty term such as LASSO for the purpose of relevant feature selection [14], and the Bayesian factor analysis (GBFA) framework [15]. So far, the iCluster method [11] and its updated versions have been widely applied to analyze many genomics datasets that cover a variety of cancers such as for glioblastoma [16] and esophageal cancer [17].

In our opinion, longitudinal gene expression data can be regarded as a special case of multiple omics data or multiple-view data integration [18], with the expression profiles at a single time point from the same individuals being viewed as one of multiple data. In this article, we show that direct utilization of an integrative analysis algorithm such as the iCluster method [11] to longitudinal gene expression data is feasible by analyzing psoriasis lncRNA expression profiles.

## Methods and materials

### Experimental data

A microarray dataset [8] whose accession number is GSE85034 in the Gene Expression Omnibus (GEO) database (https://www.ncbi.nlm.nih.gov/geo/) was used to identify relevant lncRNA to predict the response status of individual patients to immune treatments. There were 179 arrays in this experiment, which involved the gene expression profiles of 30 patients with moderate to severe psoriasis at the baseline with both non-lesion skins and lesion skins, and at weeks 1, 2, 4, and 16. Of these 30 patients, half were treated with adalimumab (ADA) and the other half were treated with methotrexate (MTX). Of note, one patient who was on the ADA arm had no expression level measured at week 16 given his/her PASI score already achieved a reduction of 75% at the week 4 (thus had been discharged). In Table S1, demographic characteristics of the 30 patients were presented.

The pre-processed data (ready for reanalysis) that were quantile normalized were downloaded from the GEO database. By matching the gene symbols of lncRNAs in the GENCODE (https://www.gencodegenes.org/) database (version 32) to those of genes annotated by the Illumina HumanHT-12 V 4.0 bead chips, 662 unique lncRNAs were identified, upon which the downstream analysis was carried out.

### iCluster

The integrative clustering method (iCluster) proposed by Shen et al. [11] uses a joint latent variable model to combine multiple omics data together and then cluster samples into distinct groups. Briefly, in the model T genomic data matrix *X*_it_ (*t*=1, …, *T*) are related to a set of latent variables *Z*_i_ (*i*=1, …, *n*) in the following way,

$$ {X}_{it}={W}_t{Z}_i+{\varepsilon}_{it} $$

here, *W*_t_ represents the coefficient of gene *g* for data type *t* (here, for time point *t*) and *ε*_*it*_ is the error terms. Conditioned on the latent variable *Z*_i_, *X*_it_ are independent from one another. The correlations of different genomic data for the same people are modeled with these latent variables. In the iCluster model, an expectation-maximization (EM) algorithm is used for parameter estimation. By using a soft-threshold method to continuously shrink the coefficients of non-informative values toward 0’s, the iCluster method [11] simultaneously accomplishes data integration, dimension reduction, feature selection, and then divides samples into different subgroups according to the latent variables. Readers are referred to the original article for a detailed description of the iCluster method.

In this study, the iCluster/iClusterPlus method is adopted to analyze longitudinal gene expression data that involve four time points—lesional tissues at the baseline, week 1, week 2, and week 4, with the objectives of selecting important lncRNAs which can distinguish responders from non-responders to a specific immune therapy, revealing the underlying therapeutic mechanisms of the treatment and thus detecting patients who are highly likely to respond and thus benefit from the treatment as early as possible. Consequently, instead of representing multiple data types, the index *t* in the above equation corresponds to time points.

The iCluster method is essentially an unsupervised learning method whose predictive performance is usually inferior to a supervised learning method. To address this issue, by following the idea of an ensemble learning method, we randomly selected a small subset of lncRNAs (here, we set the size at 20 for a fast implementation) and performed clustering repeatedly by applying the iCluster method to the resulting subsets for 10,000 times. Of note, we disabled feature selection of the iCluster method by setting the tuning parameter *λ*’s to zero. This consideration is based on the fact that we only used a small subset of lncRNAs for each replicate. In addition, the number of clusters in iCluster was set at two given that the response status to a specific treatment is the outcome of interest.

Then, we combined the resulting lncRNA lists of learners whose accuracy is > 75%, and ranked the lncRNAs according to self-customized scores which may be used to evaluate the importance of certain lncRNAs in the overall integrated “stronger” learner. These scores were calculated by summing up a specific gene’s absolute *W*_t_ values in those “weaker” iCluster learners. For a specific gene, therefore, if |*W*_t_|=0 for (*t*=1,…*T*) then this gene would be ruled out. On the other hand, if the sum of |*W*_t_| is large enough, which may correspond to two extreme cases—either the magnitude of |*W*_t_| is very large at a single time point or two or their values are subtle at all time points but when added up together the sum is large enough, the certain gene is subject to temporal changes over time. Alternatively, the maximum of |*W*_t_| may be used to represent the importance of a certain gene. However, it would lead to a high probability of missing the latter scenario. We believe that this strategy can help obtain a stronger and more robust learner and identify core lncRNAs associated with the outcome of interest. This procedure is referred to as the iCluster ensemble hereafter, and the R codes of iCluster ensemble have been restored in the Github repository (https://github.com/windytian/icluster_ensemble-).

### Statistical language and packages

All statistical analyses were carried out in the R language, version 3.6.1 (http://www.r-project.org), with the aid of Bioconductor packages and CRAN packages. Specifically, iClusterPlus [11, 12] was used for iCluster analysis, org.Hs.eg.db for gene annotation, and EDGE for EDGE analysis [19], pheatmap [20] to generate heatmaps, locfit [21] for local regression fitting, glmnet [22] for LASSO analysis, gee (https://cran.r-project.org/web/packages/gee/gee.pdf) for fitting the GEE models, and e1071 (https://cran.r-project.org/web/packages/e1071/e1071.pdf) for support vector machine modeling.

## Results

Using the majority of voting (for each replicate), the prediction of response status for all samples was made. If the number of predicting the sample as a responder is more than that of non-responder, then the specific sample is classified as a responder (vice versa), the iCluster ensemble of 10,000 replicates resulted in an overall accuracy of 83.33%, with 5 responders being misclassified as non-responders (2 were on MTX treatment and 3 on ADA). Notably, if only the learners with accuracy >80% were considered, the final accuracy was increased slightly to 86.67%. Nevertheless, given there were only 6 leaners that met this stringent cutoff and most lncRNAs within these 6 leaners only appeared once (mostly subject to the randomness), a less stringent cutoff was chosen.

Ranking decreasingly according to the self-defined scores in the “Materials and Methods” section, we selected the first 20 lncRNAs as core genes to build up a classification model and predict the response status of a psoriasis patient to a specific immune therapy. Table 1 presents the gene symbols of the identified 20 lncRNAs on the list.

**Table 1:** The 20-lncRNA list identified by the iCluster ensemble

Gene symbol	Score used to evaluate the importance of a specific gene^a^	Biological relevance (confidence score)
LINC00936	11.07	
FAM13A-AS1	9.23	
PSMA3-AS1	6.49	
LINC00173	4.78	
PCED1B-AS1	4.73	I (0.91)
SCARNA9	3.87	I (0.18)
TMEM99	3.03	I (0.02)
H19	2.89	D (0.18)
LINC00640	2.65	
PAXIP1-AS1	2.63	
MAPKAPK5-AS1	2.02	I (0.11)
DICER1-AS1	1.92	I (0.04)
MIR600HG	1.9	
SNHG7	1.82	I (0.1)
ZMIZ1-AS1	1.79	I (0.08)
TMCC1-AS1	1.75	
CD27-AS1	1.7	D (0.71)
FRMD6-AS1	1.62	
TRHDE-AS1	1.59	
URB1-AS1	1.56	

*Note*: D, directly related to psoriasis according to the GeneCards database; I, indirectly related to psoriasis according to the GeneCards database. The confidence scores are indicative of how much evidence supports the biological relevance, with a higher value corresponding to a stronger support

^a^A self-defined score is calculated as the sum of absolute weights over the replicates of iCluster modeling, which is used to determine if a certain gene should be selected in the final model. For more details, please refer to the “Materials and Methods” section

Of the 20 lncRNAs on the list, *LINC00936* (also known as *ATP2B1-AS1*) ranked at the top. In the literature, we cannot find evidence to link this lncRNA to psoriasis. Future experimental validation of its role in psoriasis is highly desirable. However, the lncRNADisease 2.0 knowledgebase [23] suggested this lncRNA was experimentally validated to correlate with astrocytoma and computationally predicted to associate with several other cancer types. In that validation study [23], *ATP2B1-AS1* was identified as a differentially expressed gene in astrocytoma using a microarray experiment. Additionally, using the GeneCards database [24], the biological relevance of all 20 lncRNAs was evaluated and this information is also given in Table 1. Actually, only *CD27-AS1* and *H19* were indicated to directly relate to psoriasis, and seven other lncRNAs were indirectly related to psoriasis.

**Fig. 1:**
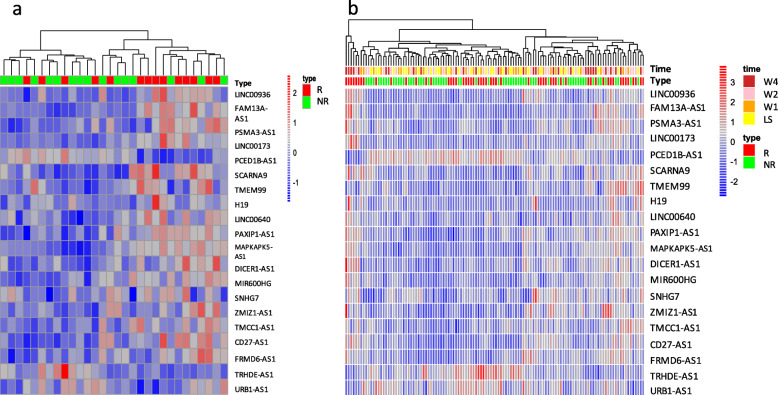
Heatmap of identified 20 lncRNAs expression profiles. **a** Heatmap of the average expression values across four time points for the 20 lncRNAs. **b** Heatmap of actual expression values at individual time points for the 20 lncRNAs. Type: response status, corresponding to the responder group and the non-responder group that were colored by red and green, respectively. From plot A, it is observed that the responders and non-responders may be clustered into several separate communities by the hierarchical clustering method. If the number of clusters was set at 2, eleven patients were misclassified, resulting in an unsatisfactory performance. In plot B, the similar pattern has been observed. Of note, the expression values have been normalized to have a mean of 0 and a variance of 1, for a clearer representation of these 20 lncRNAs. The color bars on the right side indicate the ranges of gene expression values, with red for high expression values and blue for low expression values

The heatmap of average expression values for the 20 lncRNAs across the baseline (lesional skin), week 1, week 2, and week 4 is shown in Fig. 1a, from which, it is observed that the responders and non-responders may be clustered into several separate communities by the hierarchical clustering method. When the number of clusters was set at 2, about 11 patients were misclassified, resulting in an unsatisfactory performance. Thus, we resorted to a supervised method for a more precise segmentation between the responders and the non-responders. Specifically, using support vector machine (SVM) models and leave-one-out (LOO) method (one sample has been left out, the iCluster ensemble was trained on the remaining 29 samples and then the top 20 lncRNAs were selected), the predictive accuracy of the iCluster ensemble (still, the average expression values of lncRNAs before week 4 were used to generate pseudo-genes that served as the covariates) was calculated as 80%, with 5 responders misclassified as non-responders and one non-responder as responders. Furthermore, the heatmap of actual expression values for the 20 lncRNAs at these four time points is presented in Fig. 1b, from which the similar pattern that the responders and non-responders are mixed together is observed.

To investigate the predictive capacity of the resulting 20-lncRNA list, we have randomly selected a set of 20 lncRNAs for 1000 times. Subsequently, SVM models were fit on the LOO data using the randomly selected 20 lncRNAs as predictors, and then the predictive accuracies for these 1000 replicates were calculated and averaged. The baseline accuracy of a 20-gene list is estimated as 53.47%. Therefore, the 20-gene list identified by the iCluster ensemble outperforms the randomly selected 20-gene list.

Furthermore, a comparison between the iCluster ensemble and three competing methods, namely, iCluster (using all 662 lncRNAs), LASSO [25], an ensemble with LASSO as the basic learner, GEE-based screening [26], and EDGE [19] were made. For a comparison with iCluster, the effect of ensemble can be evaluated. As expected, a single run of iCluster alone resulted in an unsatisfied performance, which is identical to that of LASSO. Even though iCluster can analyze multi-view data, its nature of being an unsupervised learning method introduced many noises.

For the GEE-based screening (the working correlation structure was fixed at the unstructured one since the time points are unequally spaced), GEE models with unstructured working correlation structure were fit for individual genes and the top 20 lncRNAs (most significant) were selected. Upon the 20-gene list, LOO support vector machine models were fit to calculate the predictive error rate, which is estimated as 33.33% and is inferior to the 20% achieved by the iCluster ensemble method.

As a specific feature-selection method to handle longitudinal data, the EDGE method (which is also a filter method) has been widely utilized. When setting the cutoff value of FDR at 0.05, 27 lncRNAs were deemed as differentially expressed genes across time between the responder group and the non-responder group by the EDGE method. Then, LOO SVM models were fit to estimate predictive accuracy of the 27-gene list, whose value is 56.67%.

Specifically, LASSO is an embedded method that identifies relevant features and constructs the final classifier simultaneously. In order to fit LASSO, the longitudinal expression profiles need to be downgraded as cross-sectional expression profiles by calculating the averages of each gene across time points. For this application, most LASSO methods select no lncRNAs at all (which corresponds to the null model), resulting in an error rate of 46.67%, which is very close to a random guess. Furthermore, we replaced iCluster with LASSO to frame LASSO-ensemble in which a LASSO logistic model was used as the basic learner to identify relevant lncRNAs among randomly selected 100 genes. Based on the sum of estimated coefficients for the 10,000 replicates, the top 20 lncRNAs were selected. Then LOO SVM models were fit to estimate predictive accuracy of the LASSO ensemble, whose value is 73.33%, presenting a substantial improvement over LASSO. The results of this comparison are presented in Table 2. Overall, iCluster-ensemble outperformed the competing methods.

**Table 2:** Comparison between iCluster-ensemble and competing methods

Method	Are feature selection and classifier construction separate	Size	Predictive error
iCluster-ensemble	Yes	20	20%
iCluster	Yes	20	46.47%
GEE-based screening	Yes	20	33.33%
EDGE	Yes	27	43.33%
LASSO	No	0.43^a^	46.67%
LASSO-ensemble	Yes	20	26.67%

^a^Since LASSO builds up the final model simultaneously with feature selection, the sizes of final model differ in single LOO runs. Here, the average of the sizes over resulting 30 LASSO models is given. Predictive error corresponds to the leave-one-out error (LOO) rate

Based on the above comparison, we concluded that the superiority of iCluster-ensemble may be due to two aspects: one is a method capable of analyzing longitudinal data and the ensemble that enables to abstract a stronger learner from weak learners. Moreover, the contribution of an ensemble may be substantially bigger, while it also addresses the drawback of iCluster being an unsupervised learning method. In addition, since the relevant biomarkers for the two treatments may differ, separate analyses stratified by treatments using iCluster-ensemble were also performed, and the results are given in Table S2.

Another application of the iCluster-ensemble procedure on a longitudinal microarray dataset of patients with multiple sclerosis was made, and the analysis results were presented in Supplementary File 1. Basically, the results (Table S3) consist with the results of the psoriasis application, namely, iCluster-ensemble performs the best among the competing methods.

**Fig. 2:**
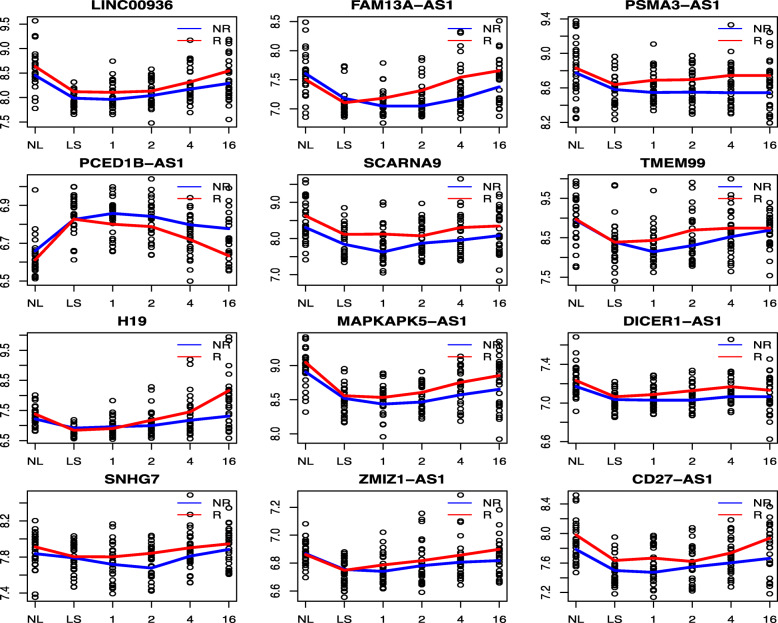
Change trajectories of 12 lncRNAs’ expression values over time stratified by the response status. In these plots, *x*-axis represents the time points measured, and *y*-axis represents the expression values. The averages of certain lncRNAs at separate time points were connected to a curve to represent their respective change trajectories over time. It is observed that for the responder group, most genes’ expression values moved back toward the expression values of non-lesional skins. This pattern became very apparent at week 4 and later. For the non-responder group, this pattern is much less apparent. NR, the non-responder group; R, the responder group; NL, non-lesional skin at the baseline; LS, lesional skin at the baseline; 1, lesional skin at week 1; 2, lesional skin at week 2; 4, lesional skin at week 4; 16, lesional skin at week 16

Using the loess (local regression) method, the change trajectories of identified lncRNAs’ expression profiles stratified by the response status were made (including the top three genes and those that were indicated to be biologically relevant to psoriasis, reducing the number of lncRNAs under consideration to 12), and are presented in Fig. 2. From this figure, we observed that for the responder group, the gaps between lesional skins (LS) and non-lesional (NL) skins had closed up as the time advanced. This pattern became apparent even at week 4. All expression changes of the identified 20 lncRNAs except *FRMDEAS1* possessed this pattern. The certain temporal change pattern over time in the responder group suggested the expression level of these lncRNAs recovered to their respective normal values; thus, these lncRNAs may have prognostic values on the response status indeed. As an example, for *H19*, the average expression value for non-lesional skin is 7.405, while it reached the plateau (the minimum) for baseline lesional skin, the average expression level turned up back and monotonically increased, even surpassed the non-lesional level and climbed up to 8.188 at week 16 in the responder group. In contrast, the U-shape in the non-responder group has much less curvature. Actually, it looks more like a horizontal line.

Lastly, the target mRNAs by these 20 lncRNAs were retrieved from the lncRNADisease 2.0 knowledgebase [23] and fed into the String software [27] for the enrichment analysis of KEGG pathways [28] and GO terms [29]. Five GO biological process terms, three GO molecular function terms, nine GO cellular component terms, and one KEGG pathway were enriched by the target mRNAs. Those enriched terms and pathways are given in Table 3. Among these enriched terms or pathways, proteasome and protein deubiquitination appeared several times. The ubiquitination-proteasome system is regarded as a key player in psoriasis, and a proteasome inhibitor to target ubiquitination pathway holds promises for treating psoriasis [28].

**Table 3:** Enriched pathways by target mRNAs of the 20-lncRNA list

ID	Description	Observed gene count	Background gene count	False discovery rate
GO: cellular component				
GO:0005839	Proteasome core complex	7	21	<0.0001
GO:0019773	Proteasome core complex, alpha-subunit complex	6	8	<0.0001
GO:0000796	Condensin complex	5	7	<0.0001
GO:0000799	Nuclear condensin complex	2	3	0.0033
GO:1904813	Ficolin-1-rich granule lumen	4	125	0.0213
GO:0043232	Intracellular non-membrane-bounded organelle	24	4005	0.0302
GO:0044444	Cytoplasmic part	43	9377	0.0302
GO:0000932	P-body	3	81	0.0475
GO:0005737	Cytoplasm	48	11238	0.0475
GO: molecular function				
GO:0004298	Threonine-type endopeptidase activity	7	21	<0.0001
GO:0005031	Tumor necrosis factor-activated receptor activity	3	25	0.0092
GO:0070011	Peptidase activity, acting on l-amino acid peptides	8	603	0.0362
GO: biological process				
GO:0007076	Mitotic chromosome condensation	5	15	<0.0001
GO:0010032	Meiotic chromosome condensation	4	5	<0.0001
GO:0016579	Protein deubiquitination	7	275	0.0099
GO:0006323	DNA packaging	6	195	0.0111
GO:0043687	Post-translational protein modification	7	365	0.0253
KEGG pathway				
hsa03050	Proteasome	7	43	<0.0001

## Discussion

As far as psoriasis is concerned, the research on its relevant lncRNA markers is really rare, explaining why in the lncRNADisease 2.0 knowledgebase [23], a search on lncRNAs that have been experimentally validated to associate with psoriasis returned nothing. Focusing on the 20 lncRNAs identified by the iCluster ensemble, the lncRNADisease 2.0 knowledgebase [23] suggested that only *H19* was predicted to be associated with psoriasis by some computational methods. Overall, the literature mining and the lncRNA canonical knowledgebase search found limited valuable information on the roles that this 20-lncRNA signature may play in combating psoriasis.

It is worth pointing out that there are several limitations in this study. First, the sample size is not very large. Stratified by the treatment arms, there were only 15 patients in each stratum. Given these two treatments may differ in terms of underlying therapeutic mechanisms and targeted molecular markers or pathways, separate analyses stratified by treatment arms were conducted and the results are presented in the Additional file 1.

Second, this study had not been carried out in a specific platform for lncRNAs. As a result, some crucial lncRNAs for psoriasis may be absent in this analysis. For example, psoriasis associated non-protein coding RNA induced by stress (*PRINS*) which has been shown to exhibit the highest expression levels in non-psoriatic skin lesions and play an important role in pathogenesis of psoriasis does not belong to the 662 lncRNAs under consideration in this study. As aforementioned, lncRNA investigations on psoriasis are rare, let alone here, we considered a longitudinal study. To the best of our knowledge, the present study is among the first effort to explore the association between lncRNAs and psoriasis using longitudinal gene expression data.

Lastly, the predictive performance of the identified 20-lncRNA list was not validated on an independent dataset, resulting in a potential overestimation. This is due to the shortage of an independent dataset that has same or similar objectives and study design, in addition to a decent sample size. A large longitudinal lncRNA study is needed to reveal the therapeutic mechanism of an immune treatment for psoriasis and thus predict the response status as early as possible, from the perspective of lncRNAs.

## Conclusions

In addition to being viewed as a gene set [30–32], longitudinal gene expression profiles can be regarded as a special case of multiple data sets/multiple-view data. Consequently, many integrative methods that combine those multiple omics data together such as [31, 32] may be adopted directly to analyze longitudinal data. Direct utilization of existing methods saves time and resources to develop new statistical methods to specifically handle longitudinal big data.

In this study, a well-known integrative clustering method, namely, the iCluster method was used repeatedly to devise an ensemble for longitudinal microarray data analysis, with the objective of identifying relevant lncRNAs to predict response status of psoriasis patients to immune therapies. Using the iCluster ensemble and longitudinal lncRNA expression values during the early period of treatments for patients with psoriasis, our analysis highlighted 20 lncRNAs that may hold predictive values for distinguishing between the responders and the non-responders to immune treatment. Further investigation on these 20 lncRNAs to reveal comprehensively how they function in concert triggered by immune treatment to fight psoriasis is warranted.

## Supplementary Information

**Additional file 1: Supplementary File 1–**Another application of the iCluster ensemble procedure on multiple sclerosis data, and separate analyses stratified by treatments for psoriasis data. **Table S1**—The clinical and demographic characteristics of psoriasis patients in the longitudinal microarray experiment. **Table S2—**Relevant lncRNAs identified by separate analyses. **Table S3—**Comparison between iCluster-ensemble and competing methods for the multiple sclerosis application.

## Data Availability

Pre-processed data (Accession #: GSE85034) were downloaded from the GEO database (https://www.ncbi.nlm.nih.gov/geo/).

## Abbreviations

iCluster: Integrative clustering
lncRNA: Long non-coding RNA
LS: Lesional
NL: Non-lesional
GEO: Gene expression omnibus
GO: Gene ontology
MEG3: Maternally expressed gene3
ceRNA: Competing endogenous RNA
WGCNA: Weighted gene correlation network analysis
PRINS: Psoriasis-associated non-protein coding RNA induced by stress
