# Supplementary material for: An ensemble of the iCluster method to analyze longitudinal lncRNA expression data for psoriasis patients
Source: Hum Genomics. 2021 Apr 20;15:23. doi: 10.1186/s40246-021-00323-6 (PMC8056592; doi:10.1186/s40246-021-00323-6)
Supplement: Supplementary file 1 — Additional file 1: Supplementary File 1–Another application of the iCluster ensemble procedure on multiple sclerosis data, and separate analyses stratified by treatments for psoriasis data. Table S1—The clinical and demographic characteristics of psoriasis patients in the longitudinal microarray experiment. Table S2—Relevant lncRNAs identified by separate analyses. Table S3—Comparison between iCluster-ensemble and competing methods for the multiple sclerosis application. [file 40246_2021_323_MOESM1_ESM.docx]

**Stratified by treatments**

In **Table S1**, the demographic characteristics of 30 psoriasis patients are given. It is worth pointing out that for each individual treatment, the ratios of responders to non-responders are not 1:1, with patients responding better to the ADA treatment.

Then, we carried out separate analyses for each treatment to identify treatment-specific relevant lncRNAs using the iCluster-ensemble procedure. As expected, while several relevant lncRNAs are common for these two treatments, there are also lncRNAs specific for individual treatments. The identified lncRNAs and their biological relevance to psoriasis are presented in **Table S2**.

**An application to multiple sclerosis data**

Furthermore, the iCluster-ensemble procedure was applied to another longitudinal microarray data and a comparison with other competing methods, namely, LASSO, iCluster, and Lasso-ensemble was made, with the objective of exploring iCluster-ensemble deeper.

The microarray experiment dataset used in this application is GSE24427 [1]. In this experiment, longitudinal gene expression profiles from 25 German RRMS patients treated with recombinant IFN- β-1b (250 µg every other day) for 2 years were collected. After restricting the responder category to patients whose first relapse time was more than 5 years nine responders and nine non-responders were considered for the downstream analysis. For more details on the MS longitudinal dataset, our previous work [2] is referred.

Then, the statistical modeling was performed using the four methods/procedures. Same as the psoriasis application, LOO predictive errors were calculated to evaluate and compare the performance of these methods/procedures. Of note, only LASSO can select relevant genes and build up the final model simultaneously. Therefore, for the other three methods, a support vector machine model was used to construct the final classifier.

The results are given in **Table S3**, from which it is found that iCluster-ensemble performs slightly better than LASSO-ensemble and ranks the top among the four methods. This is in consistent with the results of psoriasis application.

**References**

[1] Hundeshagen A, Hecker M, Paap BK, Angerstein C, Kandulski O, Fatum C, Hartmann C, Koczan D, Thiesen H-J, Klaus Zettl UK. Elevated type I interferon-like activity in a subset of multiple sclerosis patients: molecular basis and clinical relevance. Journal of Neuroinflammation 2012, 9(1):140.

[2] Jin T, Wang C, Tian S. Feature selection based on differentially correlated gene pairs reveals the mechanism of IFN-ß therapy for multiple sclerosis. PeerJ 2020, 8:8812.

**Table S1. Demographic characteristic of psoriasis patients in the longitudinal microarray experiment.**

| Patient | Time points | Treatment | Status |
| --- | --- | --- | --- |
| 1 | NL, LS, W1, W2, W4, W16 | MTX | NR |
| 2 | NL, LS, W1, W2, W4, W16 | MTX | R |
| 3 | NL, LS, W1, W2, W4, W16 | ADA | R |
| 4 | NL, LS, W1, W2, W4, W16 | ADA | R |
| 5 | NL, LS, W1, W2, W4, W16 | MTX | NR |
| 6 | NL, LS, W1, W2, W4, W16 | ADA | R |
| 7 | NL, LS, W1, W2, W4, W16 | MTX | NR |
| 8 | NL, LS, W1, W2, W4, W16 | ADA | R |
| 9 | NL, LS, W1, W2, W4, W16 | MTX | NR |
| 10 | NL, LS, W1, W2, W4 | ADA | R |
| 11 | NL, LS, W1, W2, W4, W16 | MTX | NR |
| 12 | NL, LS, W1, W2, W4, W16 | ADA | R |
| 13 | NL, LS, W1, W2, W4, W16 | MTX | NR |
| 14 | NL, LS, W1, W2, W4, W16 | ADA | R |
| 15 | NL, LS, W1, W2, W4, W16 | ADA | NR |
| 16 | NL, LS, W1, W2, W4, W16 | MTX | NR |
| 17 | NL, LS, W1, W2, W4, W16 | MTX | NR |
| 18 | NL, LS, W1, W2, W4, W16 | ADA | R |
| 19 | NL, LS, W1, W2, W4, W16 | ADA | NR |
| 20 | NL, LS, W1, W2, W4, W16 | MTX | NR |
| 21 | NL, LS, W1, W2, W4, W16 | MTX | R |
| 22 | NL, LS, W1, W2, W4, W16 | ADA | R |
| 23 | NL, LS, W1, W2, W4, W16 | ADA | R |
| 24 | NL, LS, W1, W2, W4, W16 | MTX | R |
| 25 | NL, LS, W1, W2, W4, W16 | MTX | NR |
| 26 | NL, LS, W1, W2, W4, W16 | ADA | NR |
| 27 | NL, LS, W1, W2, W4, W16 | ADA | NR |
| 28 | NL, LS, W1, W2, W4, W16 | MTX | R |
| 29 | NL, LS, W1, W2, W4, W16 | MTX | NR |
| 30 | NL, LS, W1, W2, W4, W16 | ADA | NR |

Note: R: responder; NR: non-responder; NL: non-lesional skin at the baseline; LS: lesional skin at the baseline; W1: at week 1; W2: at week 2; W4: at week 4; W16: at week 16; ADA: adalimumab; MTX: methotrexate. Since patient 10 already achieved a 75 % reduction in PASI score at week 4, his/her gene expression profiles at week 16 were not measured.

**Table S2. Relevant lncRNAs identified by separate analyses.**

| MTX | | ADA | |
| --- | --- | --- | --- |
| Symbol | Biological relevance  (confidence score) | Symbol | Biological relevance  (confidence score) |
| GLIDR |  | **MIR205** | I (1.47) |
| LOC729950 |  | LINC01137 | I (0.04) |
| **MIR205** | I (1.47) | EGOT | D(0.72) |
| STX17-AS1 |  | SCARNA9 | I (0.15) |
| **TMEM99** | I (0.02) | LINC01102 |  |
| AFAP1-AS1 | I (0.17) | **KCNQ1OT1** | I (0.1) |
| LOC401463 | I (0.51) | **TMEM99** | I (0.02) |
| ZNF674-AS1 |  | MAPKAPK5-AS1 | I (0.09) |
| **KCNQ1OT1** | I (0.1) | **LINC00936** |  |
| CEBPA-AS1 |  | PAXIP1-AS1 |  |
| LINC01565 | I (0.25) | PSMA3-AS1 |  |
| SNHG9 | I (0.09) | HOTAIR | I (1.99) |
| LINC00323 |  | LINC00909 | I (0.13) |
| ST7-AS1 | I (0.05) | H19 | D (0.13) |
| SNHG15 | I (0.7) | CD27-AS1 | D (0.72) |
| LINC01018 |  | SNHG7 | I (0.8) |
| LOC340357 | I (1.46) | LOC400043 |  |
| LINC00636 |  | **LINC01554** | I (0.14) |
| **LINC00936** |  | MAFG-AS1 |  |
| **LINC01554** | I (0.14) | LINC00640 |  |

Note: D: directly related to psoriasis according to the GeneCards database; I: indirectly related to psoriasis according to the GeneCards database. The confidence scores are indicative of how much evidence supports the biological relevance, with a higher value corresponding to a stronger support.

**Table S3. Comparison between iCluster-ensemble and competing methods for the multiple sclerosis application**

| Method | Size | Predictive error |
| --- | --- | --- |
| iCluster-ensemble | 10 | 11.11% |
| iCluster^1^ | 10 | 33.33% |
| LASSO* | 6.83 | 38.89% |
| LASSO-ensemble | 10 | 16.67% |

Note: Since LASSO builds up the final model simultaneously with feature selection, the sizes of final model differ in single LOO runs. Here, the average of the sizes over resulting 18 LASSO models is given. Predictive error corresponds to the leave-one-out error (LOO) rate. ^1^In order to speed up the iCluster modeling, the edge method was used firstly to select the differentially expressed genes (DEGs) across time and then the iCluster analysis was carried out upon these DEGs.
